# A novel technique for precoagulation of blood vessels during saline-immersion colorectal endoscopic submucosal dissection: flushing forced method

**DOI:** 10.1055/a-2499-9964

**Published:** 2025-01-14

**Authors:** Hiromitsu Ban, Tatsuma Nomura, Takashi Toyonaga

**Affiliations:** 1 469844 Department of Gastroenterology, Omi Medical Center, Kusatsu, Japan; 2 Department of Gastroenterology, Suzuka General Hospital, Suzuka, Japan; 3 Division of Gastroenterology, Department of Internal Medicine, Kobe University Graduate School of Medicine, Kobe, Japan

Endoscopic visibility is maintained during saline-immersion endoscopic submucosal dissection (ESD), and this technique is widely used for management of colorectal and duodenal tumors. We previously proposed the F1–10 method for the precoagulation of vessels under gaseous conditions 1 2 . However, under saline immersion, the same high frequency setting cannot stabilize the current, resulting in insufficient precoagulation. Recently, an endoscopic water irrigation pump (EIP2; Erbe Elektromedizin GmbH, Tübingen, Germany) was reported to remove bubbles generated during ESD under saline-immersion conditions 3 . Thus, to ensure precoagulation under saline-immersion conditions, we developed the “flushing forced method” using the EIP2.

The flushing forced method uses an endoscopic water irrigation pump (EIP2) and an electrosurgical knife (Flush Knife BTs; FUJIFILM Corp., Tokyo, Japan) connected by an extension tube. In this technique, pressing the foot pedal automatically causes water to flow out of the base of the needle-knife. Thus, even at a high radiofrequency output, electrical discharges are less likely to occur at the knife tip, and a precoagulation similar to the F1–10 method can be obtained under saline conditions ( Fig. 1 ). The high-frequency setting (ERBE VIO 3) for precoagulation was set to the coagulation mode (forced coagulation mode effect, 6.5).

**Fig. 1:**
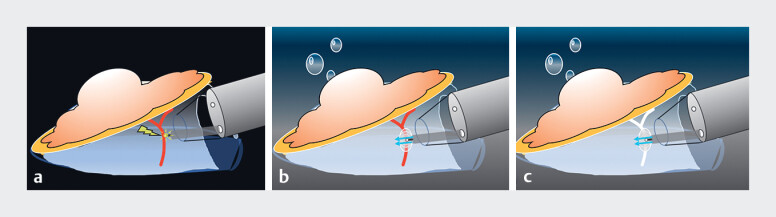
Schema of precoagulation under saline-immersion conditions using the flushing forced method. **a** Coagulation using the usual forced coagulation mode under carbon dioxide gas causes a strong electrical spark. **b** Precoagulation using the flushing forced method allows appropriate precoagulation to be achieved because the saline flow is continuous and does not cause an electrical spark, resulting in gradual coagulation. **c** Blood vessel after precoagulation using the flushing forced method.

The patient in our case was a 76-year-old man with a 60-mm colonic tumor in the cecum ( Fig. 2 , Video 1 ). ESD was performed by the flushing forced method. If vessels were visible in the submucosa, precoagulation was performed on the vessels using the flushing forced method and was continued until the vessels stopped pulsating. Dissection of blood vessels after precutting was performed in the high-cut mode.

**Fig. 2:**
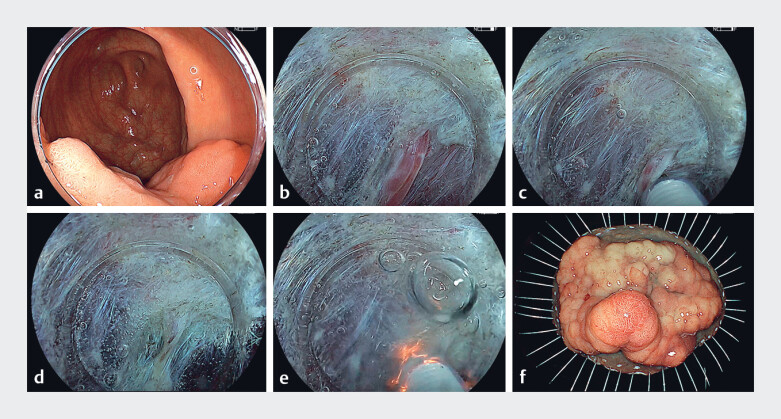
Colorectal endoscopic submucosal dissection using the flushing forced method. **a** A 60-mm laterally spreading tumor (granular mixed type) in the cecum. **b** Vessels in the submucosa. **c** Precoagulation using the flushing forced method. **d** Vessels that have changed to white after precoagulation. **e** Dissection of vessels after precoagulation using the high-cut mode. Unlike the forced mode, the high-cut mode is capable of generating an electrical spark even under saline-immersion conditions. **f** Pathology results of the resected specimen showed intramucosal carcinoma.

A novel technique for precoagulation of blood vessels in saline-immersion colorectal endoscopic submucosal dissection: the flushing forced method.Video 1

The flushing forced method is thus a novel technique for safe precoagulation of blood vessels, even under saline immersion.

Endoscopy_UCTN_Code_TTT_1AQ_2AD_3AD

## Notes

Endoscopy E-Videos https://eref.thieme.de/e-videos
*E-Videos* is an open access online section of the journal *Endoscopy* , reporting on interesting cases and new techniques in gastroenterological endoscopy. All papers include a high-quality video and are published with a Creative Commons CC-BY license. Endoscopy E-Videos qualify for HINARI discounts and waivers and eligibility is automatically checked during the submission process. We grant 100% waivers to articles whose corresponding authors are based in Group A countries and 50% waivers to those who are based in Group B countries as classified by Research4Life (see: https://www.research4life.org/access/eligibility/ ). This section has its own submission website at https://mc.manuscriptcentral.com/e-videos .
